# Near infrared surface-enhanced Raman scattering based on star-shaped gold/silver nanoparticles and hyperbolic metamaterial

**DOI:** 10.1038/s41598-017-05939-0

**Published:** 2017-07-14

**Authors:** Chih-Hsien Lai, Guo-An Wang, Tsung-Kai Ling, Tzyy-Jiann Wang, Po-kai Chiu, Yuan-Fong Chou Chau, Chih-Ching Huang, Hai-Pang Chiang

**Affiliations:** 1 Department of Electronic Engineering, National Yunlin University of Science and Technology, Yunlin, 64002 Taiwan; 2 Institute of Optoelectronic Sciences, National Taiwan Ocean University, Keelung, 20224 Taiwan; 3 Institute of Electro-Optical Engineering, National Taipei University of Technology, Taipei, 10608 Taiwan; 4 Instrument Technology Research Center, National Applied Research Laboratories, Hsinchu, Taiwan; 5 Centre for Advanced Material and Energy Sciences, Universiti Brunei Darussalam, Tungku Link, Gadong, BE1410 Negara, Brunei Darussalam; 6 Department of Bioscience and Biotechnology, National Taiwan Ocean University, Keelung, 20224 Taiwan; 7 Institute of Physics, Academia Sinica, Taipei, 11529 Taiwan

## Abstract

It is desirable to extend the surface-enhanced Raman scattering (SERS) from the conventionally used visible range into the infrared region, because the fluorescence background is lower in the long-wavelength regime. To do this, it is important to have a SERS substrate suitable for infrared operation. In this work, we report the near infrared SERS operation based on the substrates employing star-shaped gold/silver nanoparticles and hyperbolic metamaterial (HMM) structure. We first fabricate the SERS substrate in which nanoparticles are separated from a silver film by a thin dielectric layer. Performance of the SERS substrate is investigated with a 1064-nm excitation source. Compared with similar silver film-based substrates employing respectively gold and silver spherical nanoparticles, it is found that, Raman intensity scattered by the substrate with star-shaped nanoparticles is 7.4 times stronger than that with gold nanoparticles, and 3.4 times stronger than that with silver nanoparticles. Following this, we fabricate the SERS substrate where the star-shaped nanoparticles are deposited over a HMM structure. The HMM structure comprises three pairs of germanium-silver multilayers. Further experimental result shows that, with the star-shaped nanoparticles, the HMM-based substrate yields 30% higher Raman intensity for near infrared SERS operation than the silver film-based substrate does.

## Introduction

Surface-enhanced Raman scattering (SERS) is an efficient spectroscopy technique^1–5^, which finds wide applications in biosensing^6–15^, material science^16–18^, and electrochemistry^19^. With such a powerful sensing technique, even single molecules can be detected^20–24^. The major SERS mechanism relies on the localized surface plasmon resonance (LSPR) occurring at a rough metal surface, where the localized electric field is enhanced^25^. Owing to this electromagnetic effect, Raman intensity scattered by the rough metal surface of the SERS substrate can be greatly enhanced, because the scattering is proportional to the forth power of the electric field^26^. It has been shown that the optimal SERS enhancement can be obtained if the LSPR wavelength of the substrate is located between the excitation laser wavelength and the Stokes shifted wavelength^27^. SERS study is conventionally conducted with excitation laser in the visible wavelength ranging from 400 to 800 nm. In recent years, the use of infrared wavelengths for SERS has received a lot of research interests^28–32^. One advantage of extending SERS into the infrared region is the reduction of fluorescence background. In addition, the enhancement factor of SERS increases when both wavelengths of the LSPR and the excitation laser shift to a longer regime^27, 28^.

Enhancement of the localized electric field is most pronounced around the sharp tip or narrow gap, which usually appears in nanostructures. Hence, to make an effective SERS operation, it is important to have an appropriate nanostructure on the SERS substrate, especially the wavelength of LSPR being close to that of excitation. One of the commonly employed nanostructures is the spherical metal nanoparticles, and gold and silver are the two most popular metals used^33^. Various nanoparticle shapes such as rod^34, 35^, cube^36, 37^, triangular prism^38, 39^, island^40, 41^, fiber^42^, and compass^43^ have been fabricated. Recently, the star-shaped gold/silver bimetallic nanoparticle was reported^44^. By controlling the ratio between gold and silver for synthesis, absorption wavelength (i.e., plasmon resonance wavelength) of the star-shaped gold/silver nanoparticles was shown to be tunable from visible to near infrared.

In this work, the star-shaped gold/silver nanoparticles are used to fabricate two types of substrates for infrared SERS operation. In the first type, the star-shaped nanoparticles are separated from a silver film by a thin layer of aluminium oxide (Al_2_O_3_). Such a nanoparticle-on-mirror structure^45–48^ has been shown to be an effective structure for SERS substrate. When the thickness of the dielectric layer (the Al_2_O_3_ layer) between the nanoparticles and the metal film (the silver film) is sufficiently small, the electric field in the dielectric layer is strongly enhanced owing to the coupling between the localized surface plasmons (LSPs) of the nanoparticles and the surface plasmon polaritons (SPPs) of the metal film^45, 49^. The first type of substrate is referred to as the silver film-based substrate in the following. To make a comparison, two alternative silver film-based substrates are fabricated as well by replacing the star-shaped nanoparticles with gold and silver spherical nanoparticles respectively. Raman intensities are measured for these three silver film-based substrates with a 1064-nm excitation laser. From the experimental results, it is found that, the substrate employing star-shaped gold/silver nanoparticles has the strongest Raman intensity. The intensity is 7.4 times higher than that of the substrate employing gold spherical nanoparticles, and 3.4 times higher than that of the substrate employing silver spherical nanoparticles. In the second type of substrate we fabricate, the silver film is replaced by three pairs of germanium-silver multilayers and the Al_2_O_3_ dielectric layer is replaced by a thin germanium layer. The composite structure of the germanium-silver multilayers functions as a hyperbolic metamaterial (HMM)^50, 51^, and the second type of substrate is therefore referred to as the HMM-based substrate. Further experiment indicates that, for the HMM-based substrates, the one employing star-shaped gold/silver nanoparticles still exhibits stronger Raman scattering than the ones employing gold and silver nanoparticles do under the excitation wavelength of 1064 nm. Moreover, when the star-shaped gold/silver nanoparticles are employed for both cases, Raman intensity of the HMM-based substrate is observed to be 30% higher than that of the silver film-based substrate. Our study shows that, the star-shaped gold/silver nanoparticle outperforms gold and silver spherical nanoparticles under the near infrared excitation. In addition, the substrate based on star-shaped gold/silver nanoparticles and HMM structure is highly promising for SERS study in the near infrared region.

## Results

**Figure 1:**
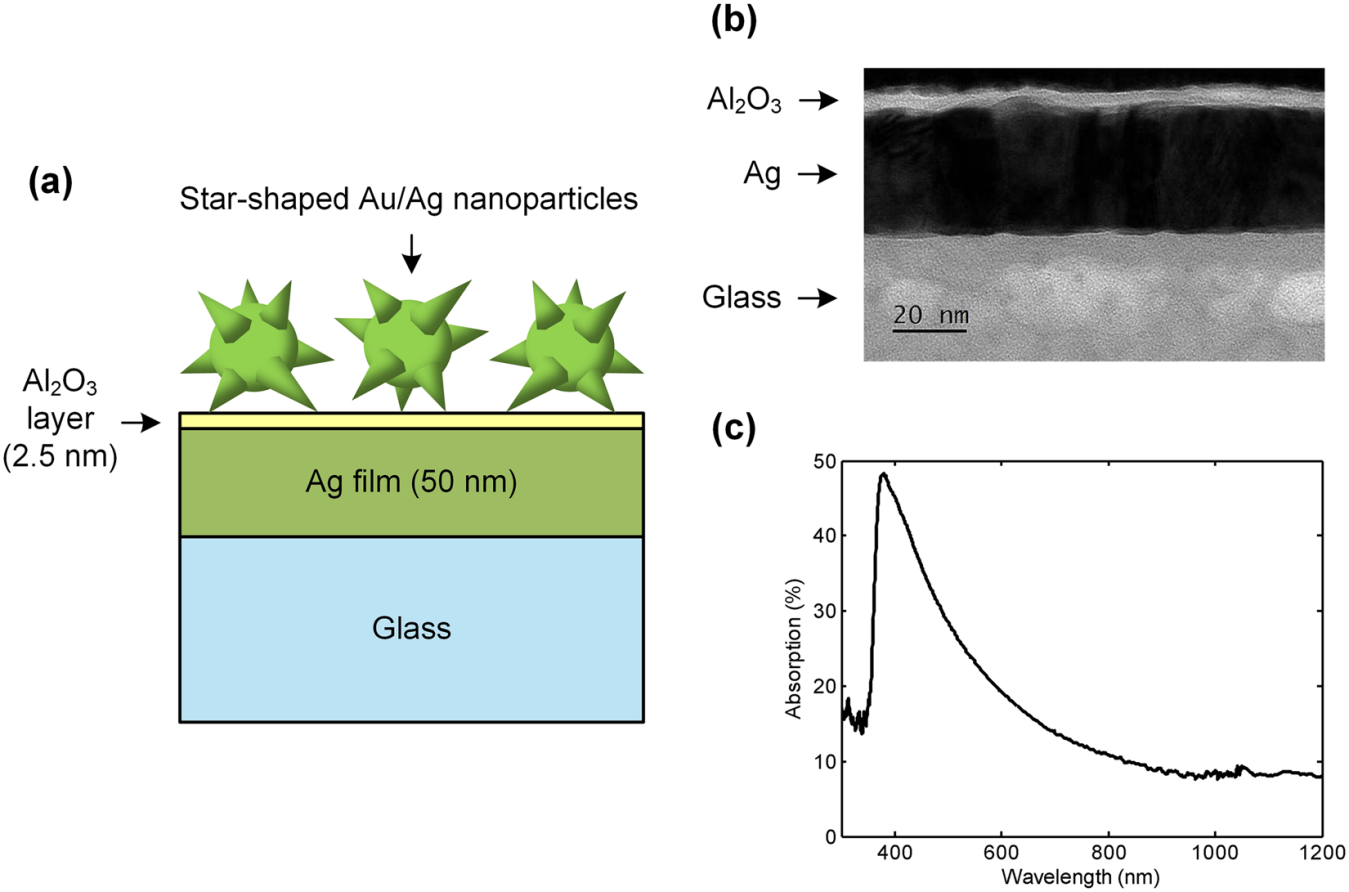
Characteristics of the silver film-based SERS substrate. (**a**) Schematic diagram of the silver film-based substrate (not to scale). Gold, silver, and star-shaped gold/silver nanoparticles are employed in this work. The diagram illustrates the case with star-shaped nanoparticles being employed. (**b**) TEM image of the bulk silver film substrate where nanoparticles are not deposited. (**c**) Absorption spectrum of the bulk silver film substrate.

Structure of the silver film-based substrate is illustrated in Fig. 1(a). Metal nanoparticles, which can be gold, silver, or star-shaped gold/silver nanoparticles, are separated from the silver film by a 2.5-nm thick Al_2_O_3_ layer. Thickness of the silver film is 50 nm. Figure 1(b) shows a transmission electron microscopy (TEM) image of the bulk substrate (i.e., without nanoparticles). In addition, absorption spectrum of the bulk substrate is shown in Fig. 1(c). From the spectrum, it is observed that the bulk substrate has an absorption peak at 380 nm, which results primarily from the silver film.

**Figure 2:**
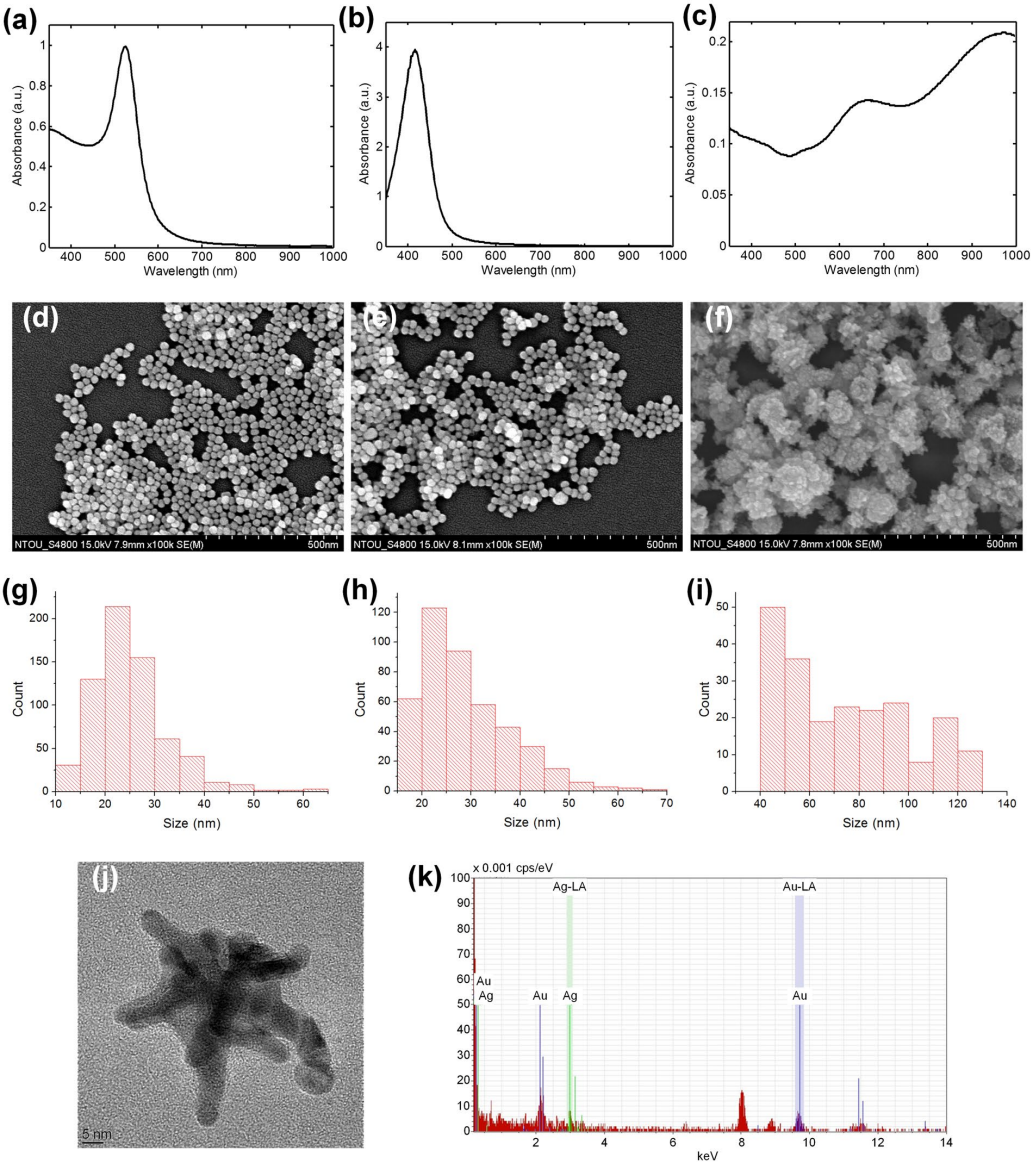
Characteristics of the metal nanoparticles. Absorption spectra of solutions containing (**a**) gold, (**b**) silver, and (**c**) star-shaped gold/silver nanoparticles. SEM images of nanoparticles deposited on the silver film-based substrates for (**d**) gold, (**e**) silver, and (**f**) star-shaped gold/silver nanoparticles. Distributions of particle size, determined from the SEM images, for (**g**) gold, (**h**) silver, and (**i**) star-shaped gold/silver nanoparticles. (**j**) TEM image of a single star-shaped gold/silver nanoparticle having a particle size (tip to tip) of 62 nm. (**k**) Elemental analysis of the star-shaped gold/silver nanoparticles by EDX.

It is also important to study the absorption spectra of gold, silver, and star-shaped gold/silver nanoparticles before deposition on the bulk substrate. By measuring solutions containing individual nanoparticles, absorption spectra of the three kinds of nanoparticles are shown in Fig. 2(a)‒(c). As can be seen, absorption peaks are 524 and 416 nm for gold and silver spherical nanoparticles, respectively. While for the star-shaped gold/silver nanoparticle, there are two absorption peaks: 664 and 974 nm. The former corresponds to resonance of the core of the star-shaped nanoparticle; and the latter, which is the dominant one, corresponds to resonance of the tips^44^. Note that the absorption wavelength of the star-shaped nanoparticles can be tuned by adjusting the Au/Ag ratio of synthesis^44^. To make the star-shaped nanoparticles suitable for infrared SERS study, these particles were synthesized with an Au/Ag ratio of 10. According to the literature, such a ratio can have the dominant absorption wavelength falling within the near-infrared region^44^, as demonstrated in Fig. 2(c). Compared with the spectra shown in Fig. 2(a) and (b), absorption of the solution containing star-shaped nanoparticles is found to be weak, and this is due to the low concentration of star-shaped nanoparticles.

Figure 2(d)‒(f) shows the images, obtained by scanning electron microscope (SEM), of the three kinds of nanoparticles deposited on the silver film-based substrates. Particle densities and sizes can be determined from the SEM images by using the image process program ImageJ. The particle densities (particles/μm^2^) are 583, 387, and 189 for gold, silver, and star-shaped nanoparticles, respectively. Distributions of particle size are shown in Fig. 2(g)‒(i) for the three kinds of nanoparticles. The average sizes in diameter are 25 nm, 29 nm, and 74 nm for gold spherical nanoparticles, silver spherical nanoparticles, and star-shaped gold/silver nanoparticles, and the corresponding standard deviations of size are 7 nm, 9 nm, and 25 nm. A TEM image of a single star-shaped gold/silver nanoparticle is shown in Fig. 2(j). The tips of the particle can be clearly observed from the TEM image. The size of this star-shaped nanoparticle is 62 nm from tip to tip. The elemental analysis of the star-shaped gold/silver nanoparticles by energy-dispersive X-ray (EDX) microscopy is provided in Fig. 2(k).

**Figure 3:**
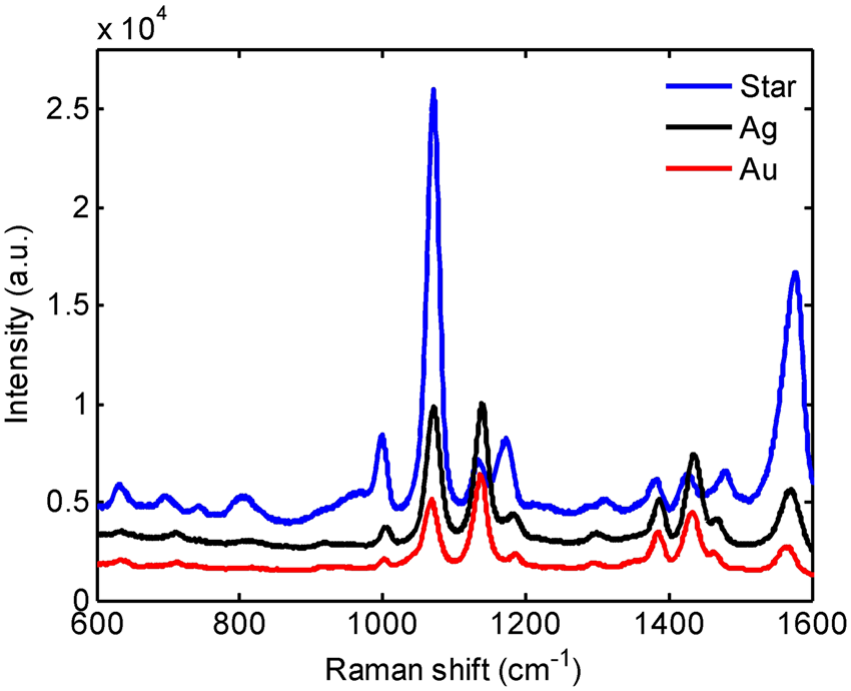
Raman spectra of 4-ATP molecule with the silver film-based substrates. Raman spectra of 4-ATP molecule with the silver film-based substrates employing gold, silver, and star-shaped gold/silver nanoparticles.

Using a 1064-nm laser as the excitation source, SERS spectra of the 4-aminothiophenol (4-ATP) molecules were respectively measured for the silver film-based substrates employing gold, silver, and star-shaped nanoparticles. Measured results are shown in Fig. 3. Clearly, the substrate with star-shaped gold/silver nanoparticles has the strongest Raman scattering intensity. Although the effect of particle size may have a little contribution^52^, this occurrence is principally attributed to the following two reasons. First, the dominant absorption peak of the star-shaped nanoparticles (974 nm) is in close proximity to the excitation laser wavelength (1064 nm), which results in a stronger LSPR. Second, nonspherical morphology of the star-shaped nanoparticles, especially the area around the sharp tips, also facilitates the enhancement of the localized electric field. Both mechanisms lead to the notable enhancement of the scattered Raman intensity. From the measured Raman spectra shown in Fig. 3, taking the Raman shift of 1073 cm^−1^ as the sampling point, the intensities (after reducing the background fluorescence) for gold, silver, and star-shaped gold/silver nanoparticles are 2744, 6012, and 20466, respectively. That is to say, the scattered Raman intensity of star-shaped gold/silver nanoparticles is 7.4 times stronger than that of gold spherical nanoparticles and 3.4 times stronger than that of silver spherical nanoparticles.

One may argue that, although the Raman intensities of gold and silver nanoparticles shown in Fig. 3 are lower than that of star-shaped nanoparticles, the intensities can be enhanced by increasing the particle size and density. For example, if the metal nanoparticle has a larger size, the absorption peak will move toward the longer wavelength regime, which might yield a stronger LSPR under the 1064-nm excitation. However, this would have little effect because it has been shown that the enhancement of SERS signal will saturate when the diameter of the particle increases^52^. While for the particle density, scattered Raman intensity is indeed proportional to the density of nanoparticles deposited on the SERS substrate. Hence, to make a fair assessment, it is helpful to calculate the intensity contribution from a single nanoparticle, which is defined as

1 $${\rm{Intensity}}\,{\rm{contribution}}=\frac{{\rm{Raman}}\,{\rm{intensity}}}{{\rm{Number}}\,{\rm{of}}\,{\rm{nanoparticles}}}$$

Here, we want to compare the intensity contributions among the three kinds of nanoparticles, and we are interested only in the relative strengths of intensity contributed by single gold, silver, and star-shaped nanoparticles. By assuming the numbers of nanoparticles within the excitation laser spot area for the three kinds of nanoparticles are proportional to the particle densities appearing in the three SEM images shown in Fig. 2(d)‒(f), without loss of generality, it is convenient to replace the denominator (number of nanoparticles) of equation (1) with the particle densities obtained from the SEM images. Substituting Raman intensities (after reducing the background fluorescence) measured at the Raman shift of 1073 cm^−1^ into the numerator of equation (1), the relative strengths of intensity contributed by single gold, silver, and star-shaped nanoparticles can be obtained as 4.7, 12.5, and 108.3, respectively. Obviously, for a single nanoparticle, the star-shaped gold/silver nanoparticle contributes a much stronger Raman intensity than the gold spherical nanoparticle and the silver spherical nanoparticle do under the 1064 nm excitation source. The contribution is 23 times larger than that of a single gold nanoparticle, and 9 times larger than that of a single silver nanoparticle.

**Figure 4:**
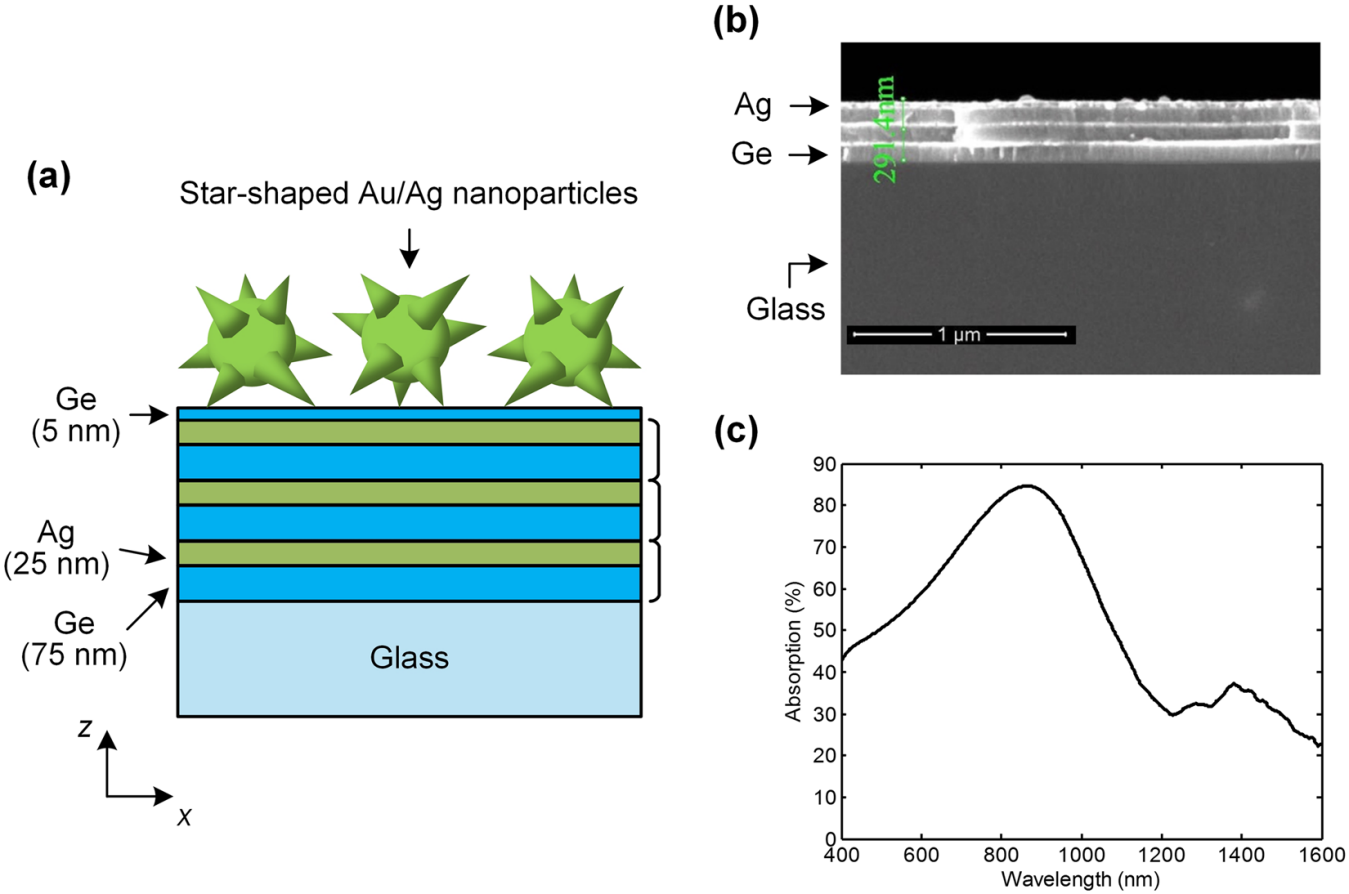
Characteristics of the HMM-based substrate. (**a**) Schematic diagram of the HMM-based substrate employing star-shaped gold/silver nanoparticles (not to scale). (**b**) SEM image of the bulk HMM substrate where the 5 nm-Ge layer and the star-shaped nanoparticles are absent. The three pairs of Ge-Ag multilayers can be clearly observed over the glass plate. (**c**) Absorption spectrum of the bulk HMM substrate.

Following this, we investigate the SERS performance for the HMM-based substrate. Figure 4(a) plots the structure of the HMM-based substrate employing star-shaped gold/silver nanoparticles. Three pairs of germanium-silver multilayers (Ge: 75 nm and Ag: 25 nm) are deposited on a glass plate. The multilayered germanium-silver composite structure is anisotropic with a uniaxial symmetry. Its effective permittivities along the directions parallel (*x*- and *y*-direction) and perpendicular (*z*-direction) to the multilayers can be estimated by using the effective medium theory^53, 54^, and the effective permittivities are obtained as *ε*_*x*_ = *ε*_*y*_ = −1.73 + 0.15*i* and ε_z_ = 25.13 + 0.03*i* at the wavelength of 1064 nm. Since (*ε*_*x*_ *ε*_*z*_) < 0, the dispersion curve is hyperbolic^53, 54^, and thus the multilayered composite structure functions as an HMM. A 5-nm thick germanium layer is placed between the composite HMM structure and the star-shaped nanoparticles. Figure 4(b) shows an SEM image of the bulk HMM substrate, in which the three pairs of germanium-silver multilayers can be clearly observed over the glass plate. Note that the purpose of employing multilayers here for the SERS substrate is not to take advantage of the hyperbolic dispersion property of HMM. Instead, it is because the absorption peak is tunable by adjusting the thicknesses of germanium and silver layers. Unlike the silver film where the wavelength of absorption peak is fixed and independent of film thickness, for the germanium-silver multilayers, thicknesses of the layers will affect the absorption wavelength of the composite structure, and this desirable property provides a chance to push the absorption peak into the infrared region. Figure 4(c) shows the absorption spectrum of the bulk HMM substrate. According to our design, the bulk HMM substrate has an absorption peak at 866 nm.

**Figure 5:**
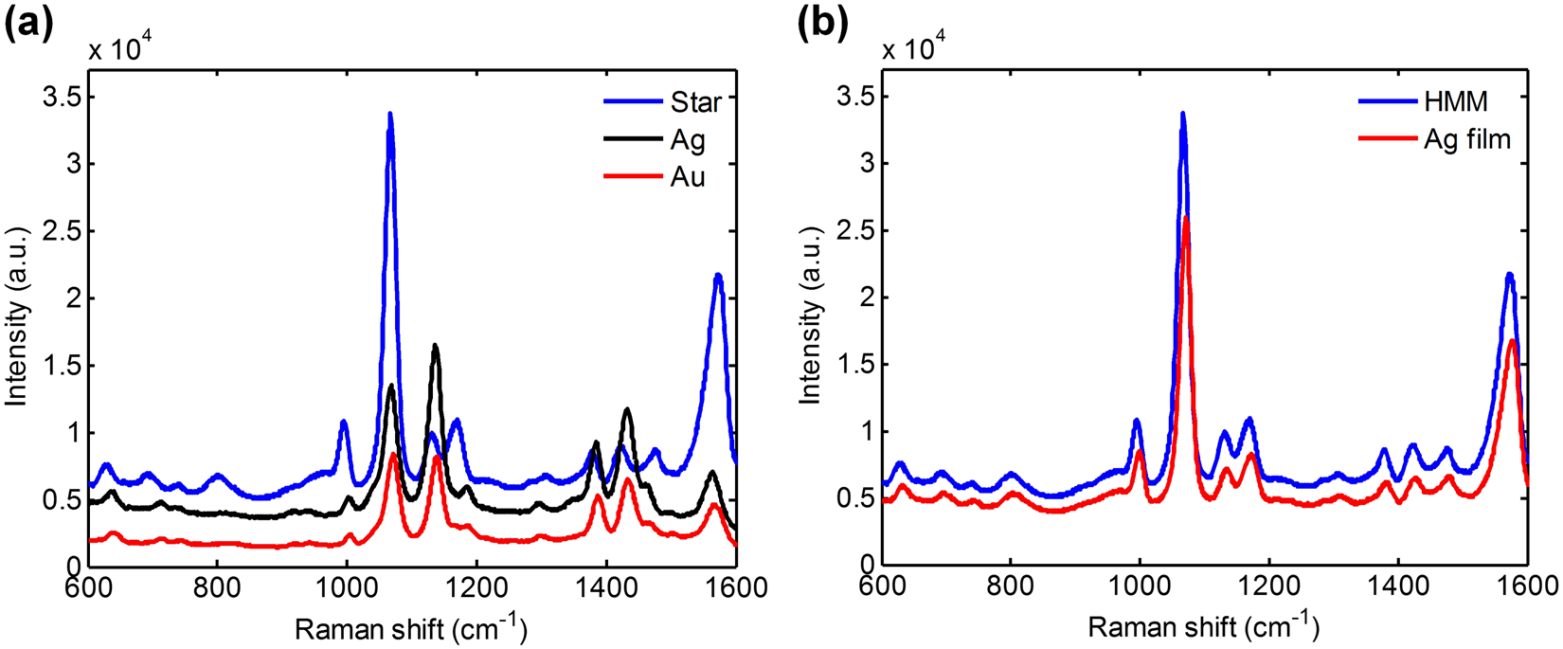
Raman spectra of 4-ATP molecule with the HMM-based substrates. (**a**) Raman spectra of 4-ATP molecule with the HMM-based substrates employing gold, silver, and star-shaped gold/silver nanoparticles. (**b**) Comparison between the HMM-based and the silver film-based substrates, both employing star-shaped nanoparticles.

With the 1064-nm laser source, SERS spectra of 4-ATP were respectively measured for the HMM-based substrates employing gold, silver, and star-shaped nanoparticles. As can be seen in Fig. 5(a), like the silver film-based case, Raman intensity of the HMM-based substrate employing star-shaped gold/silver nanoparticles is still stronger than those of the substrates employing gold and silver nanoparticles. Figure 5(b) compares the measured 4-ATP spectra for the silver film-based and the HMM-based SERS substrates, both employing the star-shaped nanoparticles. It is found that the scattered Raman intensity of the HMM-based substrate is about 30% higher than that of the silver film-based substrate. This phenomenon can be ascribed to the absorption peak (866 nm) of the HMM bulk substrate, which is closer to the excitation wavelength and thus leads to a stronger plasmon resonance.

## Conclusion

In summary, we have investigated the feasibility of employing star-shaped gold/silver nanoparticles and HMM structure for SERS study in the infrared region. Compared with gold and silver spherical nanoparticles, experimental results with a 1064 nm excitation source indicate that the SERS substrate employing star-shaped gold/silver nanoparticles has the strongest Raman intensity, which is 7.4 times higher than that employing gold spherical nanoparticles and 3.4 times higher than that employing silver spherical nanoparticles. Two reasons are responsible for the star-shaped gold/silver nanoparticle to outperform the other two kinds of spherical nanoparticles. First, its dominant absorption peaks is close to the excitation wavelength, leading to a stronger localized surface plasmon resonance. Second, the sharp tips of the star-shaped nanoparticles facilitate the enhancement of the localized electric field. While for the HMM structure, its absorption peak is also close to the excitation wavelength, and thus yields a stronger plasmon resonance and a stronger Raman intensity. In this study, the star-shaped gold/silver nanoparticles were synthesized by the chemical reduction method, which is simple, fast, and low-cost. Wavelengths of absorption peaks for the star-shaped nanoparticles are tunable by adjusting the synthesis ratio between gold and silver, and the absorption wavelength of the HMM structure can be tuned by controlling the thicknesses of the multilayers. Therefore, the substrate based on star-shaped gold/silver nanoparticles and HMM structure has the features of excellent SERS performance, rapid synthesis, and tunable absorption wavelength, which make it promising for SERS study in the infrared region.

## Methods

### Preparation of nanoparticles

Nanoparticles employed in this study were all prepared by using the chemical reduction method. To prepare the gold spherical nanoparitcles, a 50 ml 0.01% HAuCl_4_ solution was heated to boil. Then, a 1% sodium citrate solution was added quickly into the heated HAuCl_4_ solution. After a reaction period of 8 minutes, the mixed solution was put in ice bath to obtain the gold nanoparticles.

For the preparation of the silver spherical nanoparticles, a solution containing silver seed was prepared in advance by mixing 10 ml de-ionized (DI) water, 50 μl 100 M sodium citrate, 50 μl 100 mM AgNO_3_, and 600 μl 7.5 mM NaBH_4_ together in cold water bath and stirring for 30 minutes. After that, 2 ml silver seed solution was mixed with 4 ml DI water, 2 ml 50 mM sodium citrate, 2 ml 20 mM ascorbic acid, and 10 ml 0.2 mM AgNO_3_. It was then stirred over 2 hours.

Synthesis of the star-shaped gold/silver nanoparticles followed the procedure described in the previous literature^44^. First, 20 μl 10 mM HAuCl_4_ and 2 μl 10 mM AgNO_3_ (Au:Ag = 10:1) were mixed in 1 ml DI water. Then, 4 μl 100 mM ascorbic acid was added quickly, and the solution was pipetted and shaken for 20 seconds.

### Fabrication of SERS substrates

The fabrication process of the silver film-based substrate consists of depositing a 50-nm thick silver film onto a glass plate by thermal evaporation, and coating the silver film with a 2.5-nm thick Al_2_O_3_ layer by the e-gun technique. This intermediate structure (glass plate + silver film + Al_2_O_3_ layer) where nanoparticles were absent is referred to as the bulk silver film substrate.

For the fabrication of the HMM-based substrate, a DC magnetron sputter was used to deposit alternative layers of germanium (75-nm thick) and silver (25-nm thick) onto a glass plate. After three pairs of germanium-silver multilayers were fabricated, a 5-nm thick germanium layer was deposited finally. This intermediate structure (glass plate + three pairs of multilayers + germanium layer) is referred to as the bulk HMM substrate.

To prepare the final SERS substrate, the bulk silver film (or HMM) substrate was immersed in a solution mixed with 1 mL (3-Aminopropyl)trimethoxy-silane (APTMS) and 9 mL methanol for 3 hours. Then, the substrate was put into a centrifuge tube which contained the solution of desired nanoparticles (gold, silver, or star-shaped gold/silver nanoparticles), and centrifugation was performed at a centrifugal rate of 10000 rpm under 4 °C for 15 minutes. After that, nanoparticles would have been deposited on the SERS substrate.

### SERS measurement

To evaluate in the near infrared regime the performances of the SERS substrates with different kinds of nanoparticles, Raman spectra of 4-aminothiophenol (4-ATP) molecules were measured utilizing a confocal micro-Raman system. Confocal micro-Raman measurements were carried out at room temperature using a 1064 nm Nd:YAG DPSS laser as excitation source. A NA = 0.68 aspherical lens with 3 mm working distance was used for laser focusing and Raman signal collection. The spot size of the laser focused on the sample was calibrated to be about 1.2 µm. The incident laser was linear polarized and the laser power on sample surface was 88 mW. The un-polarized Raman signal was dispersed using a home-built NIR spectrometer with F/4.0, f = 200 mm, ruled grating blazed at 1 m and 600 grooves/mm, and measured with a TE-cooled CCD detector (i.e. Model No.: Andor iDus InGaAs DU491A-1.7). Concentration of the 4-ATP solution was 8 × 10^−4^ M.

### Calculation of effective permittivities for germanium-silver multilayers

Effective permittivities of the multilayered germanium-silver structure were estimated according to the effective medium theory^53, 54^, in which the effective permittivities along the directions parallel and perpendicular to the metal-dielectric boundary can be expressed as:

2 $$\begin{array}{l}{\varepsilon }_{x}={\varepsilon }_{y}=\rho {\varepsilon }_{m}+(1-\rho ){\varepsilon }_{d}\\ {\varepsilon }_{z}=\frac{{\varepsilon }_{m}{\varepsilon }_{d}}{(1-\rho ){\varepsilon }_{m}+\rho {\varepsilon }_{d}}\end{array}$$

where *ρ* is the filling ratio of metal, and *ε*_*m*_ and *ε*_*d*_ are permittivities of metal and dielectric, respectively. In this work, *ρ* = 0.25, while *ε*_*m*_ = −57.9 + 0.6*i* for silver^55^ and *ε*_*d*_ = 17.0 for germanium^56^ at 1064 nm.
